# Stop that nonsense!

**DOI:** 10.7554/eLife.04300

**Published:** 2014-09-09

**Authors:** Catherine L Jopling

**Affiliations:** 1 **Catherine L Jopling** is in School of Pharmacy, University of Nottingham, Nottingham, United Kingdomcatherine.jopling@nottingham.ac.uk

**Keywords:** microRNA, nonsense mutant, nonsense-mediated mRNA decay, APC, mutations, premature termination codon, human

## Abstract

Cells can avoid the effects of so-called ‘nonsense’ mutations by several methods, including a newly discovered mechanism driven by microRNA molecules.

**Related research article** Zhao Y, Lin J, Xu B, Hu S, Zhang X, Wu L. 2014. MicroRNA-mediated repression of nonsense mRNAs. *eLife* **3**:e03032. doi: 10.7554/eLife.03032

**Image** microRNA molecules are able to stop the production of truncated proteins

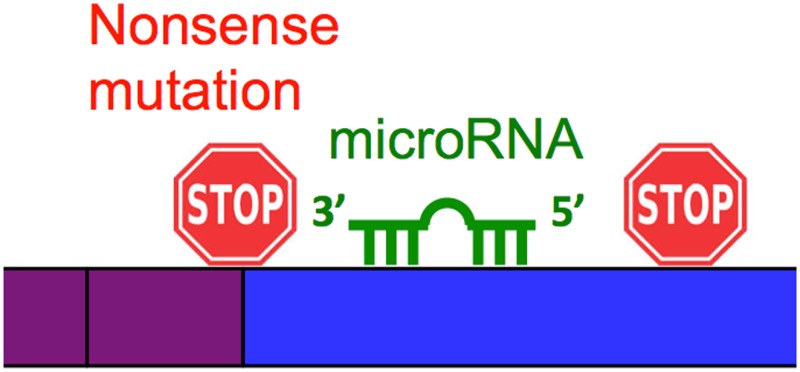

Genetic mutation is a major risk for living cells. Direct damage to DNA or errors in the processes that generate messenger RNA (mRNA) from the DNA template can introduce mutations, with potentially harmful consequences. ‘Nonsense’ mutations are particularly problematic: they are associated with many genetically inherited diseases, such as the blood disorder β-thalassaemia, and are common in cancer (Bhuvanagiri et al., 2010). To reduce the impact of these mutations, eukaryotic cells have evolved methods known as nonsense-mediated decay (or NMD for short) to destroy mutant mRNA molecules. Now, in *eLife*, Ligang Wu and co-workers at the Shanghai Institutes for Biological Sciences—including Ya Zhao as the first author—report a new mechanism of nonsense-mediated decay that is driven by microRNAs, an important family of regulatory molecules (Zhao et al., 2014).

Gene expression involves transcription, in which mRNA is copied from a DNA template, followed by translation, in which a molecular machine called the ribosome interprets the sequence of an mRNA to make the protein it encodes. Signals within the mRNA called stop codons tell the ribosome when to stop translating the mRNA (Figure 1A). Nonsense mutations introduce a stop codon ‘upstream’ of the correct signal so that translation is stopped early and a truncated protein is made. Truncations can interfere with normal protein function in various ways: if a regulatory region is lost, the protein may be over-active; the shortened non-functional proteins can also displace functional versions of the same protein from multi-protein complexes.

**Figure 1.**
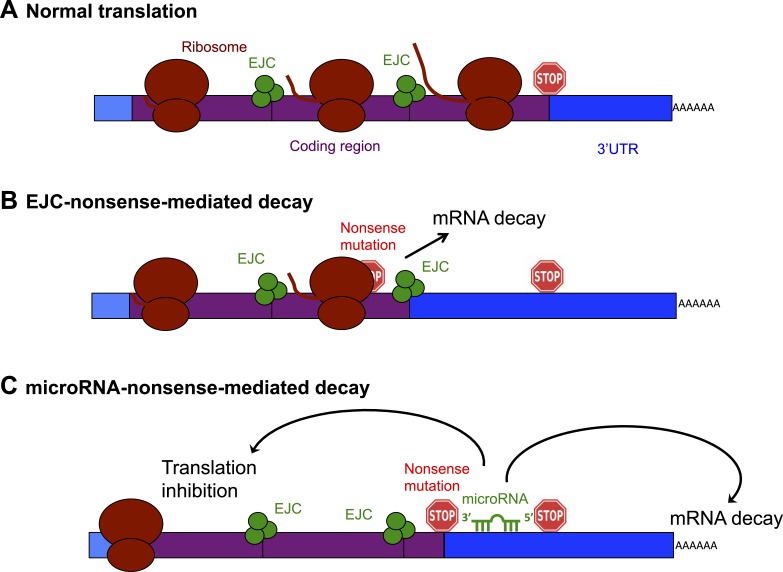
Schematic diagram showing the exon junction complex (EJC) and microRNA-driven mechanisms of nonsense-mediated decay (NMD). (**A**) In a normal mRNA molecule (blue and purple rectangle), the ribosome (brown ovals) scans along the coding region (purple), and translates a protein (brown line); this process stops when the ribosome encounters a stop codon (stop sign). The mRNA after the stop codon is called the 3′ untranslated region (3′UTR, blue), and does not encode a protein. (**B**) In EJC-nonsense-mediated decay, if a nonsense mutation is located more than 50 nucleotides upstream of an exon junction complex (EJC; green circles), this is recognized as aberrant and the mRNA is degraded. (**C**) Zhao et al. have discovered a new method of nonsense-mediated decay that depends on the regulatory actions of microRNA molecules. The coding region downstream of a nonsense mutation behaves as 3′UTR. If a microRNA binding site is present in this region, the microRNA binds to it, which inhibits translation and leads to the mRNA molecule being degraded.

The best understood method of nonsense-mediated decay relies on a process known as splicing, which removes segments of an mRNA as it is being built and sticks the remaining parts—called exons—together to make the mature mRNA. Where two exons are joined, the cell deposits proteins that form an exon junction complex (EJC) on the mRNA molecule. When this mature mRNA is being translated, the ribosome measures the distance between the EJC and the stop codon. If an EJC is found more than 50 nucleotides after (or ‘downstream’ of) a stop codon, the cell recognizes this as aberrant and destroys the mRNA (Popp and Maquat, 2013; Figure 1B).

The mRNA region downstream of the stop codon is known as the 3′ untranslated region (3′UTR). Although this region does not encode any protein, it is important for regulating the activity of the mRNA molecule. MicroRNAs are short non-coding RNA molecules (i.e., they do not encode for protein) that bind to 3′UTR sites in a sequence-specific fashion. The binding of microRNAs can inhibit the translation, and encourage the degradation, of an mRNA molecule, and is very important for controlling how and when genes are expressed in eukaryotes (Fabian and Sonenberg, 2012).

Zhao et al. reasoned that if an mRNA contains a nonsense mutation, the coding region downstream of the mutation will essentially be converted into a 3′UTR (Zhao et al., 2014). This extended 3′UTR could contain binding sites for microRNAs, which would bind to and regulate the messenger RNA molecule. Zhao et al. used a series of elegant experiments to confirm this hypothesis, and, in doing so, identified a new method of nonsense-mediated decay that is directed by microRNAs (Figure 1C). First, Zhao et al. inserted a microRNA binding site into the coding region of an mRNA. This had no effect on the mRNA, confirming the prevailing view that microRNA regulation does not occur efficiently in coding regions (Gu et al., 2009). However, when Zhao et al. introduced a nonsense codon so that the microRNA site was now located in the 3′UTR, microRNAs could bind to it, which induced mRNA decay (Zhao et al., 2014).

Zhao et al. went on to show that microRNAs also influence nonsense-mediated decay in naturally occurring mRNAs. They focused on *APC*, a tumour suppressor gene that is often found to contain nonsense mutations in colorectal cancer tumours. The *APC* gene was a particularly good choice for this study as most of its nonsense mutations occur in a hotspot within the last exon, so that they are not recognized by the exon junction complex method of nonsense-mediated decay (Miyoshi et al., 1992). However, Zhao et al. found that nonsense mutations in this region expose microRNA binding sites within the newly-formed 3′UTR, which leads to the decay of mRNA copied from the *APC* gene. Finally, Zhao et al. used reporter genes based on the breast cancer-associated tumour suppressor gene *BRCA1*, which is sensitive to the EJC form of nonsense-mediated decay, to show that the exon junction complex and microRNA mechanisms of nonsense-mediated decay can both work on the same mRNA (Zhao et al., 2014).

The findings of Zhao et al. reveal an intriguing new function for microRNAs and a new mechanism of nonsense-mediated decay. They also raise several important questions. What is the relative contribution of EJC- and microRNA-driven nonsense-mediated decay in cells? In contrast to EJC nonsense-mediated decay, microRNA regulation tends to be fairly weak (Leung and Sharp, 2010). However, multiple microRNA sites function cooperatively, so the level of microRNA nonsense-mediated decay is likely to be strongly influenced by the number and the identity of the microRNA sites revealed by a nonsense mutation. In addition, microRNA expression is highly regulated during development and in certain tissues, as well as in cancers, suggesting that microRNA-mediated nonsense-mediated decay is also likely to be tightly regulated.

Other methods of EJC-independent nonsense-mediated decay have also been discovered (Metze et al., 2013), so how does the microRNA method interact with these alternative mechanisms? By regulating the proteins involved in exon junction complex nonsense-mediated decay, microRNAs can influence how the decay proceeds (Bruno et al., 2011). Additionally, a new study has shown that microRNA binding can create a suitable target for EJC nonsense-mediated decay by changing how the ribosome reads a specific mRNA molecule (*CCR5*, which encodes a protein used by many forms of HIV to invade cells; Belew et al., 2014). Overall, it seems that the complexity of how the cell deals with nonsense mutations and how microRNAs are involved is only beginning to be revealed.
